# Multiplex PCR assays developed for neglected pathogen detection in undifferentiated acute febrile illness cases in tropical regions

**DOI:** 10.1590/0074-02760240053

**Published:** 2024-10-28

**Authors:** Leidi Carvajal Aristizabal, Karl Ciuoderis, Laura S Pérez-Restrepo, Jorge E Osorio, Juan P Hernández-Ortiz

**Affiliations:** 1 Universidad Nacional de Colombia, Global Health Institute - One Health Colombia and One Health Genomic Laboratory, Medellín, Colombia; 2 University of Wisconsin, Global Health Institute, Madison, WI, USA; 3 University of Wisconsin, School of Veterinary Medicine, Department of Pathobiological Sciences, Madison, WI, USA; 4 Universidad Nacional de Colombia, Department of Materials and Nanotechnology, Medellín, Colombia

**Keywords:** PCR, optimization, multiplex, neglected, bacteria, febrile illness

## Abstract

**BACKGROUND:**

Undifferentiated acute febrile illness (UAFI) cause by several pathogens poses a diagnostic challenge due to the similarity on the clinical manifestations across these diseases. Precise pathogen detection is vital for appropriate medical intervention, early treatment, and timely outbreak alerts regarding emerging pathogens. In tropical regions, UAFI is predominantly linked to a wide range of viral, bacterial, and parasitic infections. Hence, confirmatory laboratory tests are essential for specific pathogen identification.

**OBJECTIVES:**

Our primary goal was to develop two real-time multiplex polymerase chain reaction (PCR) assays for simultaneous detection of six neglected pathogens (*Leptospira* spp., *Rickettsia* spp., *Borrelia* spp., *Anaplasma* spp*.*, *Brucella* spp., and *Bartonella* spp.), known for causing UAFI in tropical regions.

**METHODS:**

We rigorously assessed assays parameters including: linearity, efficiency, sensitivity, and reproducibility in both singleplex and multiplex formats.

**FINDINGS:**

Our results demonstrated that these multiplex assays are reliable and sensitive methods. They showed good performance with low intra- and inter-variability (< 10%) and consistently high efficiencies (> 90%).

**MAIN CONCLUSIONS:**

These assays offer the alternative of streamlining work, reducing processing costs, and minimizing sample volume use. In conclusion, we present two dependable, user-friendly, rapid, and cost-effective methods for simultaneously detecting six neglected bacteria, as a significant laboratory tool in resource-limited tropical settings.

Undifferentiated acute febrile illness (UAFI) includes a group of diseases with challenging diagnostic attributes due to the similarity in their clinical manifestations and symptoms, often stemming from unknown sources of infection.^1^ In tropical regions like Colombia, febrile illness is a common reason for seeking medical attention.^2^ Thus, UAFI presents a significant challenge to healthcare professionals and surveillance systems due to its potential for high morbidity and mortality.^3^ The range of infectious causes for UAFI is extensive, hindering accurate and timely diagnosis and the selection of appropriate medical treatments.^4^ The majority of febrile cases in tropical areas are attributed to viral, bacterial, and parasitic infections, many of which are transmitted by animal vectors.^5^ Colombia’s geographical location, topography, and climate create appropriate conditions for vector-borne diseases.^6^ Vulnerable populations, including those residing in rural areas, marginalized communities, and conflict-affected regions, are disproportionately affected by UAFI, with many cases attributed to neglected diseases.^7^

In Colombia, priority is rightly given to the detection and surveillance of vector-borne diseases such as dengue, Zika, chikungunya, yellow fever, and malaria.^8^ However, a range of other pathogens, while less actively studied, may also be associated with UAFI, as documented in other tropical regions.^9^ Despite some investigations in Colombia, the finding of other pathogens causing UAFI remains unclear. Moreover, as the capability to detect multiple UAFI-causing pathogens simultaneously increases, the number of undiagnosed fever cases of unknown origin would be expected to decline.^3^ In this context, emerging and neglected human pathogens, including vector-borne, are gaining recognition as causes of UAFI.^10^ The public health burden that these infections posed may be underestimated, primarily due to the limited availability of multiplex detection laboratory methods, especially in low-middle income countries. Timely detection of bacterial diseases holds particular importance in guiding the targeted selection of antibiotics, mitigating inappropriate usage, and addressing antibiotic resistance concerns.^11^ With international travel on the rise, travelers may also experience UAFI after visits to tropical regions, presenting diagnostic challenges for physicians in non-tropical countries who may encounter sick travelers or migrants potentially infected with UAFI-causing pathogens.^12^ Therefore, widespread access to molecular diagnostic laboratory tools for UAFI-causing pathogens is essential.

The routine screening of individual specific pathogens by single-target molecular test in patients with UAFI presents a laborious and time-consuming process. This limitation can be effectively addressed by a molecular multiplex approach, which is capable of simultaneously detecting multiple targets from diverse pathogens.^13^ Multiplex polymerase chain reaction (PCR) offers several advantages including: time savings, enhanced efficiency, cost-effectiveness, and discriminating sensitivity, making it an attractive option for screening and monitoring pathogens.^14^ It has been widely used in the diagnosis of various infectious diseases caused by bacteria, fungi, parasites, and viruses.^13^ However, to establish a multiplex PCR assay as a standard diagnostic test, its performance and potential cross-reactivity must be rigorously evaluated.^15^

Therefore, the goal of this study was to conduct a comprehensive evaluation and assessment of the performance of two multiplex PCR assays used for the universal detection of six neglected bacteria potentially associated with UAFI in tropical regions. The work involved the development and evaluation of two distinct PCR assays including the evaluation of PCR assays for the individual detection of single-target pathogens (singleplex), and the evaluation of multiplex PCR assays. With the deployment of these multiplex PCR methods, a more precise and efficient detection of neglected bacterial pathogens in biological specimens becomes achievable. Furthermore, the presented methods have the potential for application in facilities conducting UAFI laboratory screening and may serve as tools for the development of new assays for infectious disease surveillance.

## MATERIALS AND METHODS

*Control specimens* - Control samples were used as three distinct plasmids engineered, each designed to cover a specific region of interest corresponding to the target pathogen for molecular detection [see Supplementary data (Table I)]. These plasmids were subsequently synthesized by Integrated DNA Technologies (Coralville, Iowa, USA). Upon receiving the synthesized plasmids were diluted to a concentration of 8 ng/µL. The approximate number of copies present in each plasmid was calculated utilizing Avogadro’s number (NA) and the following formula: Copy number/µL = [NA (plasmid concentration in ng/µL)] / (plasmid length x 10^9 x average weight of a base pair).

*Institutional review board statement* - The study was reviewed and approved by the Institutional Review Board of the Corporacion para Investigaciones Biologicas (CIB).

*Optimization of singleplex and multiplex PCR assays* - Primers and probes used in this work were described elsewhere [Supplementary data (Table II)]. Each set of primers was in-silico analyzed by Primer-BLAST tool^16^ using databases in National Center for Biotechnology Information (NCBI). Singleplex PCR condition was optimized according to the specific conditions of each primers set. After singleplex optimization, assays with similar characteristics were selected and two independent assays for multiplex real-time TaqMan PCR detection were set: Assay 1 included primers and probes for detection of *Leptospira* spp., *Rickettsia* spp., *Borrelia* spp., while Assay 2 included the primers and probes for detection of *Anaplasma* spp., *Brucella* spp., *Bartonella* spp. Then, each multiplex PCR was optimized and evaluated.

All real-time TaqMan PCR assays (singleplex and multiplex assays) were optimized to ensure appropriate amplification. To select the best amplification/cycling conditions, two different concentrations of the primers (0.2 µM and 0.6 µM) and the probes (0.1 µM and 0.25 µM) were tested. Optimized annealing temperature (ranging from 50ºC-60ºC) was evaluated using a gradient BioRad CFX96 thermal cycler (BioRad, Hercules, California). PCR reactions were carried out in a total volume of 12.5 μL per reaction using iTaq DNA polymerase kit (BioRad, Hercules, California). PCR reactions were run under the following conditions: 95ºC for 3 min, followed by 40 cycles of 95ºC for 15 s, 50 to 60ºC for 40 s. The best PCR condition for each assay (singleplex and multiplex) was used for the assay evaluation and performance evaluation (specificity, limit of detection, and linearity range).

*Sensitivity and specificity of PCR assays* - The analytical sensitivity and efficiency of the PCR assays (singleplex and multiplex) were determined using eight ten-fold serial dilutions of each control specimen (reference plasmid) and tested in triplicate. Not only was each dilution added in triplicate, but to have a greater number of replicates, the assay was performed twice at different times. The last dilution in which all the replicates were positive was considered as the assay limit of detection (LOD). A linear regression analysis was conducted using Python programming language (Python Software Foundation, v.3) to obtain the assay linear regression y = ax + b with a for the slope and b for the intercept. Assay amplification efficiency was calculated according to the following formula: E (%) = [10^(-1/slope)^] - 1.^15^ Amplification efficiency represents the amount of PCR product increase after each cycle. An ideal reaction reaches efficiency close to 100%. Higher E can indicate amplification of non-specific products or a pipetting error in the serial dilution. Lower E can also indicate a pipetting error in the serial dilution, poor primer design or non-optimal reaction conditions.

Specificity of multiplex PCR assays was tested using pathogens such as dengue virus, malaria, and other pathogens by in-silico testing using Primer-BLAST^16^ tool. For this, setting parameters included: a PCR product size of 70 to 1000 bp, sequences with two or more mismatches to the primers were ignored and Database of Refseq representative genomes for Bacteria (taxid:2) was selected. The other parameters were used as default.

*Inter-assay reproducibility and intra-assay repeatability of PCR assays* - Evaluation of intra- and inter-assay variability was conducted using eight ten-fold serial dilutions of each control specimen (reference plasmid) and tested in duplicate within the same run (intra-assay) and in independent trials on two different days (inter-assay) by two different operators. For singleplex PCR, each target gene was evaluated individually, while for multiplex PCR a mixture of specific pathogen molecular target was evaluated simultaneously.

*Data analysis* - The bias of each PCR assay was evaluated by calculating the coefficient of variation (CV). Variations within-run and between-trials was calculated as the percentage of the ratio of standard deviation and the average of the PCR (Ct) values. Acceptance of bias was set to 10% or less difference between the lowest value and the highest value.

*Data availability statement* - Data supporting this work are available within the reported results and supplementary information. Additional data are available upon reasonable request to the corresponding author.

## RESULTS

*Optimization of singleplex and multiplex real time PCR assays* - The standardization process began with singleplex PCR for each target, allowing the selection of the most optimal amplification conditions for individual pathogens. Subsequently, the multiplex PCR assays were conducted, with conditions grouped based on the optimal settings identified in the singleplex tests and considering cross-reactivity analysis. The final concentrations of primers and probes, as well as the optimal PCR cycling conditions for the multiplex detection of *Leptospira* spp*.*, *Rickettsia* spp*.*, and *Borrelia* spp*.*, as well as for the multiplex detection of *Anaplasma* spp*.*, *Brucella* spp., and *Bartonella* spp., are detailed in the Supplementary data (Table II).

*Analytical sensitivity and specificity of PCR assays* - The LOD for each assay was determined to assess analytical sensitivity. In the singleplex PCR assay, the LOD values were 159.5, 228.33, and 22.83 for *Leptospira* spp*.*, *Rickettsia* spp*.*, and *Borrelia* spp*.*, respectively. In the multiplex PCR assay, the LOD values were 159.5, 228.33, and 22 for *Leptospira* spp., *Rickettsia* spp., and *Borrelia* spp., respectively. For *Anaplasma* spp., *Brucella* spp., and *Bartonella* spp., the LOD values in the singleplex PCR assay were 22.79, 159.5, and 22.83, respectively, while in the multiplex PCR assay, the LOD values were 227.91, 159.5, and 228.33, respectively. A summary of the PCR performance is presented in Table.

**TABLE:** Comparison of sensitivity, limit of detection (LOD), linearity (R^2^) and efficiency of real-time multiplex and singleplex PCR taqMan assays for detection of *Leptospira* spp*.*, *Rickettsia* spp., *Borrelia* spp*.*, *Anaplasma* spp., *Brucella* spp. and *Bartonella* spp.

Assay	Target	AT	Singleplex			Multiplex			CV (%)
			R^2^	Efficiency (%)	LOD (DNA copies)	R^2^	Efficiency (%)	LOD (DNA copies)	
1	*Leptospira* spp*.*	50ºC	0.998	92.0	159.5	0.928	93.1	159.5	0.84
	*Rickettsia* spp*.*		0.995	91.6	228.33	0.963	91.3	228.33	0.23
	*Borrelia* spp*.*		0.998	94.5	22.83	0.983	94.2	22.83	0.22
2	*Anaplasma* spp*.*	60ºC	0.996	93.4	22.79	0.997	94.9	227.91	1.13
	*Brucella* spp*.*		0.993	101.8	159.5	0.923	97.6	159.5	0,87
	*Bartonella* spp*.*		0.997	97.2	22.83	0.959	98.4	228.33	2.98

AT: annealing temperature; LOD: limit of detection; R^2^: coefficient of determination; CV: coefficient of variation.

To assess linearity and efficiency, serial dilutions of a reference plasmid were used in the multiplex PCR reactions. The crossing point values of each dilution were plotted against the logarithm of the concentration expressed as DNA copy number, revealing a linear relationship spanning from 5.7 x 10^7^ to 57.4 copies/uL. It was observed that there was a slight reduction in LOD for *Anaplasma* spp. and *Bartonella* spp. when comparing singleplex and multiplex PCR detections. Additionally, the efficiency of amplification was optimal for both multiplex PCR assays, with values falling within the range of 90% to 110% (Table). Although variations in PCR efficiencies were noted when comparing singleplex and multiplex PCR assay results, these differences were not statistically significant, and all targets were detected with optimal efficiencies (> 90%) in either PCR configuration (singleplex and multiplex).

**Fig. 1:**
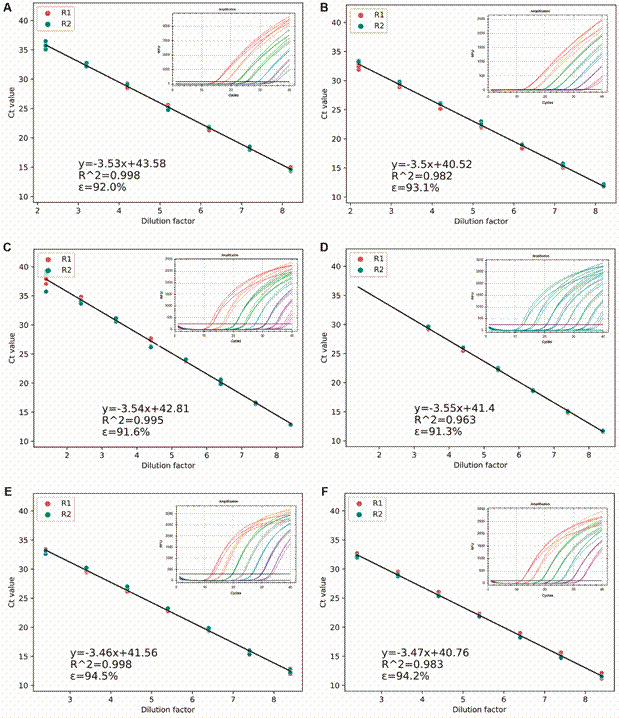
standard curves of singleplex and multiplex polymerase chain reaction (PCR) assays for detection of three neglected bacteria. Equation of the standard curve, efficiency (ε) and linearity (R^2^), as well as the real-time PCR amplification curves are shown. Each test was done twice, adding in each case each of the dilutions of the positive control in triplicate. Assays are shown as Assay 1 (R1) and Assay 2 (R2). (A) *Leptospira* spp. singleplex. (B) *Leptospira* spp*.* multiplex. (C) *Rickettsia* spp*.* singleplex. (D) *Rickettsia* spp. multiplex. (E) *Borrelia* spp. singleplex. (F) *Borrelia* spp. multiplex.

**Fig. 2:**
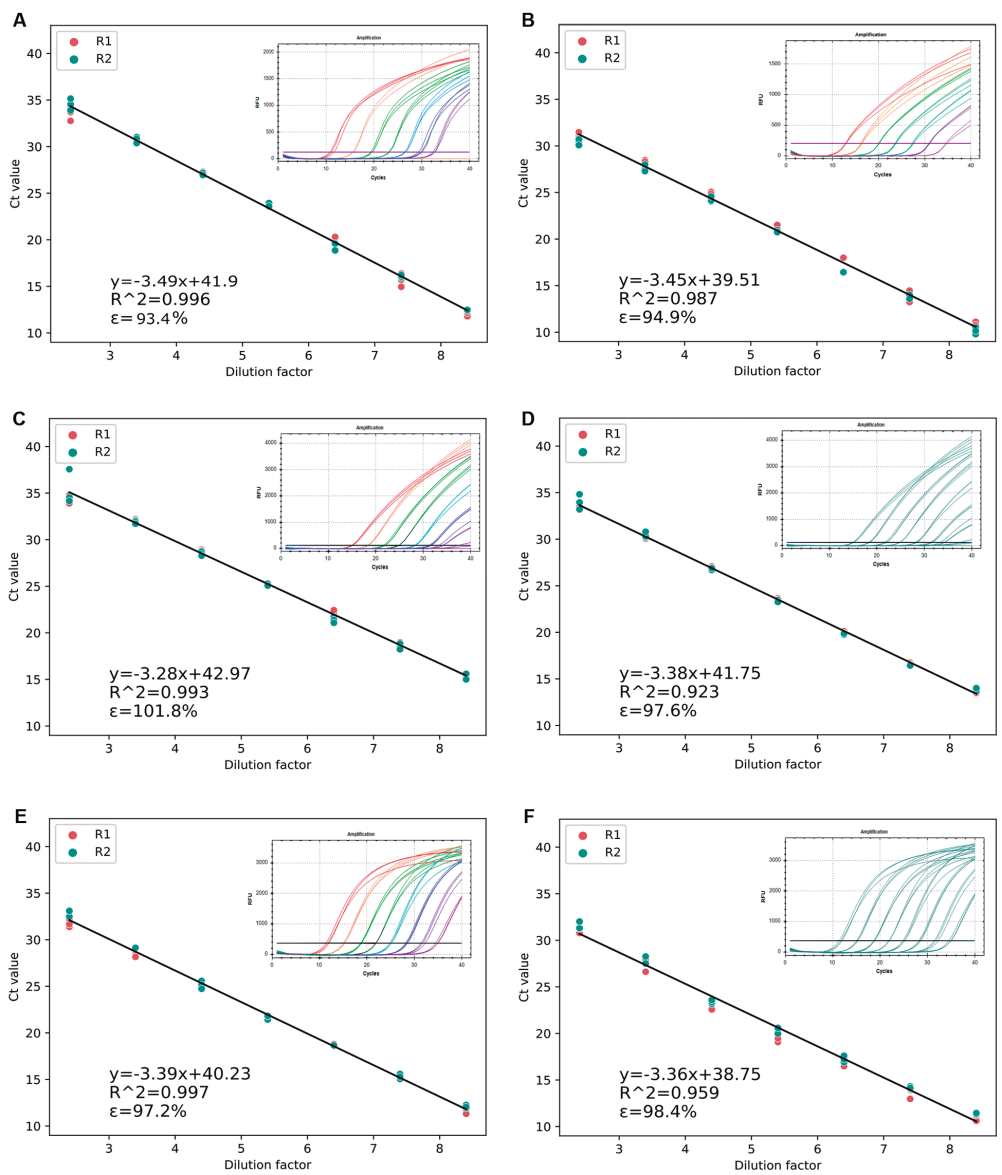
standard curves of singleplex and multiplex polymerase chain reaction (PCR) assays for detection of three neglected bacteria. Equation of the standard curve, efficiency (ε) and linearity (R^2^), as well as the real-time PCR amplification curves are shown. Each test was done twice, adding in each case each of the dilutions of the positive control in triplicate. Assays are shown as Assay 1 (R1) and Assay 2 (R2). (A) *Anaplasma* spp*.* singleplex. (B) *Anaplasma* spp. multiplex. (C) *Brucella* spp*.* singleplex. (D) *Brucella* spp*.* multiplex. (E) *Bartonella* spp*.* singleplex. (F) *Bartonella* spp*.* multiplex.

Furthermore, the PCR linearity was compared between singleplex and multiplex PCR assays for each target pathogen. The coefficient of determination (R^2^) obtained from the standard curve was used to evaluate differences. As depicted in Figs 1-2, the linearity was optimal (> 0.9) for all targets in both PCR assays (singleplex and multiplex). R^2^ values for *Rickettsia* spp., *Anaplasma* spp., and *Brucella* spp. varied between assays, but these differences were not statistically significant. The CV between PCR assays (singleplex and multiplex) was less than 5% for all targets. Additional results are summarized in Table.

Following *in-silico* analysis, both multiplex PCR assays demonstrated a remarkable specificity of 100% for detecting members of the target bacterial groups [see Supplementary data (Table III)]. No instances of positivity to other bacteria were observed during testing, thereby confirming the absence of cross-reactivity.

*Inter and intra-assay variations* - The real-time TaqMan PCR assays exhibited excellent repeatability and reproducibility. Intra-assay CV values fell within the range of 0.05% to 8.71%, while inter-assay CV values ranged from 0.06% to 6.27%, for both the singleplex and multiplex PCR assays [refer to Supplementary data (Table IV)].

## DISCUSSION

This study presents the optimization of two multiplex real-time PCR assays capable of simultaneously amplifying DNA from various bacterial species, including *Leptospira* spp., *Rickettsia* spp., *Borrelia* spp., *Anaplasma* spp., *Brucella* spp., and *Bartonella* spp. To the best of our knowledge, this research represents the first attempt to consolidate sets of primers into two multiplex PCR assays targeting six neglected pathogens known to cause AUFI in tropical regions. The results demonstrate the specificity of these assays, supported by in-silico analysis confirming that the primer sets did not cross-react with each other or with other bacteria or other pathogens. Nevertheless, it is important to note that evaluation of assay performance was not performed using clinical specimens such as real-world conditions.

Acute febrile illness is attributed to a diverse array of pathogens, including neglected bacterial species. Hence, the development of rapid, specific, and sensitive diagnostic tools to accurately identify and differentiate among these pathogens is imperative. Molecular techniques such as PCR assays offer reliability, speed, and precision in bacteria detection. However, many PCR-based diagnostic tools are designed for the individual identification of pathogen species. While a limited number of studies have reported the development of multiplex PCR tests for detecting neglected bacteria, none have encompassed the simultaneous detection of six groups of neglected bacterial species with public health significance.

Throughout the evaluation of these multiplex real-time TaqMan PCR assays, we explored the possibility of any loss in sensitivity or efficiency when compared to singleplex PCR detection. The results indicate that the assays in this study exhibited excellent linearity (R^2^ > 0.99), efficiency (> 90%), and an acceptable CV (< 10%) for all target pathogens. While the assays demonstrated optimal efficiency, sensitivity, and repeatability within this study, further verification steps are warranted to confirm their performance under real-world conditions, including clinical specimen testing.

The multiplex real-time TaqMan PCR assays optimized in this study have proven reliable and highly sensitive in detecting the target pathogens. Given the substantial cost and labor associated with individual molecular screening for multiple pathogens simultaneously, our ability to concurrently detect six pathogens using two independent PCR assays offers an efficient strategy. This approach requires fewer reagents and less labor, providing valuable results in resource-limited scenarios. Therefore, we have successfully optimized two multiplex PCR assays capable of diagnosing six neglected diseases in a single tube without compromising assay sensitivity. The implementation of these assays in real-world conditions would greatly benefit clinical and research applications in both human and veterinary medicine, as well as enhance global health surveillance efforts.
