# Supplementary material for: Multiplex PCR assays developed for neglected pathogen detection in undifferentiated acute febrile illness cases in tropical regions
Source: Mem Inst Oswaldo Cruz. 2024 Oct 28;119:e240053. doi: 10.1590/0074-02760240053 (PMC11520662; doi:10.1590/0074-02760240053)
Supplement: Supplementary file 1 [file 1678-8060-mioc-119-e240053-s.pdf]

TABLE I

Table showing reference sequences of bacterial species used for plasmid synthesis in this study

Plasmid	Target	Sequence included in the plasmid synthesized	Length (bp)	Target gene and position (bp)	Target reference sequence
A	*Leptospira* spp.	GGTTGCGCAAATTACCCAATCCTGACACAGGGAGGTAGTG<br>ACAAGAAATAACAATACAGGGCTTATAGTCTTGTAATTGGA	1947	16S ribosomal RNA (1382- 1578)	MN062732.1
	Brucella spp.	ATGATGGGGATATAAATCCTTCCCAGAGTACCAATTGGAG<br>GGCAAGTCTGGAGGGTCAGATACCGTCGTAGTCCTAACCA<br>TAAACTATCATCAAGTGATTCGATCATACGAGACATTAAAA<br>CTGAGTACCCGCATTACAAGCCGGCTAGAGATAACCCGGG<br>AACTTGATAAGTTCTGTAACGCCTATTTGATCTTGCTCTTG<br>GTGGGGTTTACCATGCGTAATTTGTTACCAACTTACCGGT<br>GCGCTCTTATCTGAAGATGCAGTTGGTTGTACTGCTGGTC<br>CTAAGGGCCTCACTGTAGCGATTAGTAAGCCTTATGGGTC<br>ACCAGAGATCACAAAGGATGGCTACAAGGTAATGAAGAGT<br>ATAAAGCCTGAAGAACCACTGGCTGCCGCTATAGCGAGCA<br>TCATTACTCAGAGTGCTTCTCAGTGTAATATGGTATGCAGT<br>TTGATCGCGGATATCTTTCGCCTTACTTTGTTACAAATGCT<br>GAAAAAATGCTGGTGGAATTTGAAAATCCATACATATTCCT<br>TACTGAAAAGAAGATTAATCTTGTACAAAGCATTTTACCAA<br>TATTAGAAAACGTTGCGAGAGCTGGCAGACCATTGCTCAT<br>CATAGCTGAAGATGTTGAAGGTGAAGCTCTTAAGGTACCT<br>ACAGAAGAAGTCCCGGCAAACTCTGTGCCAGCAGCCGCG<br>GTAATACGGAGGGGGCAAGCGTTGTTCGGAATTATTGGG<br>CGTAAAGGGCACGTAGTGGACTAGTAAGTTAAAAGTGAAA<br>TACCAAAGCTTAACTTTGGAGCGGCTTTTAATACTGCTAGA<br>CTAGAGGTCGAAAGAGGATAGCGGAATTCCTAGTGTAGA<br>GGTGAAATTCGTAGATATTAGGAGGAACACCAGTTGCGAA<br>GGCGGCTATCTGGTTCGATACTGACACTGAGGTGCGAAAG<br>CGTGGGGGAGCAAACAGGATTAGATACCCTGGTAGTCCA<br>CGCTGTAAACGATGAGTGCTAAAAGATACATGAGCAAAAT<br>CTCAACTTTATTAGAAGAGAAGCATTTATTAGATAAAAAGC<br>CAATGCATGCTTTTACAGTATGAAAATTGGTGATTTATAAT<br>AACTTAGCAAATCGTAAAGTGAAAACAGACGATAAATCATT<br>CAAGTTTCTGACCTATCAGCTTTCGAGTATCTTGGTATGTA<br>TTTAGCAATGATGTCCTTTGAAGTGTTTTTTACTTTATTGTA<br>ATAAAGCATATTCAGGACTTTTACTTTGAGAAAATTAGAGT<br>GTTTCTAGCAGGCCGACGCTTTGAATACTGCAGCATGGAA<br>TAATAAGTTTCGGGAGGCAGCAGTTAAGAATCTTGCTCAA<br>TGGGGGCAACCCTGAAGCAGCGACGCCGCGTGAACGAAG<br>AAGGTCTTCGGATTGTAAAGTTCAGTAAGCAGGGAAAAAT<br>AAGCAGCAATGTGATGATGGTACCTGCCTAAAGCACCGGC<br>TAACTACGTGCCAGCAGCCGCGGTAATACGTATGGTGCAA<br>GCGTTGTTTCGGTCTTTGTGGGCGGCTATCCGACGGGCGC<br>AATCTCGGAACTGGCCATCTCGAACGGCCATGCTGAAGAG<br>CTTGGAATGCAGCCTGCAAAAATTAACACACCAGCATCAC<br>CTAACTGGAGTAACTGGAGCTCACTAACTGGAGCACAAGC<br>TTCATGGACATTGAGAGTTCAAGTAGGTGCAAATCAAGAT<br>GAAGCAATTGCTGTACCAATGATGCATTTGAAAGCTTGGC<br>ATTGCCTGGCAAATTGGCAGATTGGCAGATTGTGCGTCTA<br>AAGTGGCAGATTGTGCGTCTAAAGCGTCTAAAAACCCGGC<br>TGAGAGGGAGATTTATATTGTAGAGGGTGATTCTGCTGGA<br>GGAAGTGCTAAAATGA		BL chromosome 1 (1603- 1637)	CP022875.1
B	*Rickettsia* spp.	TCATTCATTAGTGAATGAAAGATTACACTATTTATTTCAAAC	493	gltA (11-176)	CP004889.1
	Borrelia spp.	CTTTTGTAGCTCTTCTCATCCTATGGCTATTATGCTTGCGG<br>CTGTCGGTTCTCTTTCGGCATTTTATCCTGATTTATTGAATT<br>TTAAGGAAGCAGATTACGAACTTACTGCTATTAGAATGATT		glpQ (197- 306)	CP000993.1
	Bartonella spp.	GCTAAGATACCTACCATCGCCGGAATTGGACCTTGGATAC<br>CCCAAATCATTATTAATGGGCAAATCACAGGTCTTATAAGT<br>TTAGCTCATAAATATAACATGCAGGTTTATCCTTACACGTT<br>TAGAATTGATGCATTGCCTTCATATGTACTGGCTTACCAGC<br>TATGGTAATAAATGGACAATGAAATAAACTCATTGGACCC<br>GGGGGCGGTAGCGGAAGCACGTCTCGCTGCTTAATGTAG<br>TGTGAATGTTTCAAACTAAGCCTTAAATCGTCGCAGATTTA<br>AGCGGGGTTCGAAGGCACCTGGCAACAGAAGCCTTCACTTTG		G1712 chromosome (328- 481)	CP091505.1
C	*Anaplasma* spp.	TGATTATGAGTGACAGCGTACCTTTTGCATAATGGGTCAG<br>CGAGTTAATCTGTTGAGCAAGCTTAAGCCGTGTTAAGTTTA<br>ACGGATTAGACCCGAAACCAAGTGATCTAGCCATGGCCAG<br>GTTGAAGGTGTAGTAAAGTACATTGGAGGACCGAACCTGT<br>TACTGTTGCAATAATGACAGGGTTGGTAGGTCGTAAATCC<br>CGGTCACCTTGGTAGCCACTATAGGTGGGTCTTAAGAGAA<br>GAAGATTCCTCTTGTGCCTGCGGCGAGACCGCGCACGGT<br>CCACAGGTTTGACCAATAGGGTCACAGGGAAGGACCCTG<br>GACCCTGCCCTTCCCGGTGGGGTGGGAAATGCATGGGGC<br>CACCCAGCTCCGCGGCGGCCTCAGATTAATAGCTCTTTCT<br>TGATTTCTTGGATGGTGATGCATGGCCGTTTTTAGTTCGTG<br>AATATGATTTGTCTGGTTAATTCCGATAACGAACGAGATCT<br>TAACCTGCTAATTAGCGGT	499	23S ribosomal RNA (11- 164)	NR_076399.1

CC BY

TABLE II

Table showing sequences of primers and probes used in this studys, as well as conditions for multiplex PCR assays

Assay	Cycling conditions	Target	Description	Final concentration	Sequence 5' - 3'	Reference
1	95°C for 3 min; 40 cycles of 95°C for 15 s and 50°C for 40 s.	*Leptospira* spp.	Forward	0.22 µM	CGGGAGGCAGCAGTTAAGAA	(1)
			Reverse	0.22 µM	AACAACGCTTGCACCATACG	
			Probe	0.22 µM	FAM-TGAAGCAGCGACGCCGCGTG	
		Rickettsia spp.	Forward	0.22 µM	GTGAATGAAAGATTACACTATTTAT	(2)
			Reverse	0.22 µM	GTATCTTAGCAATCATTCTAATAGC	
			Probe	0.22 µM	CY5-CTATTATGCTTGCRGCTGTYGGTTC	
		Borrelia spp.	Forward	0.22 µM	CCTTGGATACCCCAAATYAT	(2)
			Reverse	0.22 µM	GGYAATGMRTCAATTCTAAA	
			Probe	0.22 µM	HEX-ATGGRSAAATSACAGGTCTTA	
2	95°C for 3 min; 40 cycles of 95°C for 15 s and 60°C for 40 s.	*Anaplasma* spp.	Forward	0.3 µM	TGACAGCGTACCTTTTGCAT	(2)
			Reverse	0.3 µM	GTAACAGGTTCGGTCCTCCA	
			Probe	0.15 µM	CY5-GGATTAGACCCGAAACCAAG	
		Brucella spp.	Forward	0.4 µM	TCTTTGTGGGCGGCTATCC	(3)
			Reverse	0.4 µM	CCGTTCGAGATGGCCAGTT	
			Probe	0.2 µM	FAM-ACGGGCGCAATCT	
		Bartonella spp.	Forward	0.4 µM	GCTATGGTAATAAATGGACAATGAAATAA	(3)
			Reverse	0.4 µM	GCTTCTGTTGCCAGGTG	
			Probe	0.2 µM	HEX-CGTCGCAGRTYTAAGCGGGGT	

TABLE III

Table showing a matrix of in-silico analysis of the primer's specificity in this study.
Results are shown as the number of target hits after primer-BLAST analysis

Targets	Number of hits (percentage) against the target							Specificity
	Total	*Leptospira* spp.	*Rickettsia* spp.	*Borrelia* spp.	*Anaplasma* spp.	*Bartonella* spp.	*Brucella* spp.	
Leptospira spp.	71	71 (100%)	0	0	0	0	0	100%
Rickettsia spp.	18	0	18 (100%)	0	0	0	0	100%
Borrelia spp.	12	0	0	12 (100%)	0	0	0	100%
Anaplasma spp.	12	0	0	0	12 (100%)	0	0	100%
Bartonella sp	23	0	0	0	0	23 (100%)	0	100%
Brucella sp	8	0	0	0	0	0	8 (100%)	100%

TABLE IV

Table showing inter and intra-assay variations for molecular detection of bacteria in singleplex and multiplex formats in this study

Assay configuration	Dilution	Bacterial species											
		Leptospira spp.		*Rickettsia* spp.		*Borrelia* spp.		*Anaplasma* spp.		*Brucella* spp.		*Bartonella* spp.	
		Intra-assay CV(%)	Inter-assay CV(%)	Intra-assay CV(%)	Inter-assay CV(%)	Intra-assay CV(%)	Inter-assay CV(%)	Intra-assay CV(%)	Inter-assay CV(%)	Intra-assay CV(%)	Inter-assay CV(%)	Intra-assay CV(%)	Inter-assay CV(%)
Singleplex	10^{-1}	2.56	0.27	0.35	0.57	3.82	0.25	2.31	0.56	0.75	0.31	2.8	1.42
	10^{-2}	0.51	0.36	1	0.2	2.41	0.33	4.56	0.88	0.54	1.4	0.69	1.76
	10^{-3}	0.6	1.31	0.54	1.58	0.54	1	0.39	2.21	0.88	3.03	0.5	0.19
	10^{-4}	0.66	0.96	0.86	0.07	0.89	0.85	0.71	0.72	0.05	0.52	0.15	1.03
	10^{-5}	1.54	0.77	0.69	3.39	0.63	1.16	0.13	0.51	0.45	0.77	0.81	1.69
	10^{-6}	0.5	0.07	0.25	0.43	0.53	1.2	0.3	1.11	0.62	0.35	0.62	0.18
	10^{-7}	1.33	0.44	0.94	1.48	1.18	0.06	2	1.82	1.41	1.94	1.34	1.14
	10^{-8}	-	-	1.98	0.22	-	-	1.11	1.22	-	-	0.83	0.65
Multiplex	10^{-1}	2.09	0.45	1.16	0.3	4.77	0.23	0.71	1.57	1.67	5.53	2.54	2.65
	10^{-2}	0.63	1.76	1.14	0.24	1.41	2.84	0.84	0.63	4.91	1.03	4.4	2.92
	10^{-3}	0.22	2.57	0.17	0.1	0.05	2.71	0.53	0.72	0.1	6.27	3.12	0.62
	10^{-4}	0.52	2.14	0.34	0.38	0.21	1.26	0.35	0.69	0.94	1.87	3.71	2.01
	10^{-5}	1.68	0.99	1.19	0.5	0.99	1.24	0.06	0.65	0.45	2.07	1.89	0.92
	10^{-6}	0.76	1.26	1.01	0.53	0.74	1.2	0.52	0.45	0.39	2.09	2.54	0.87
	10^{-7}	1.65	1.93	1.19	1.09	1.2	0.56	0.47	1.4	0.58	2.23	0.89	1.87
	10^{-8}	8.71	0.08	0.91	1.39	1.21	1.27	-	-	-	-	-	-

REFERENCES

1. Waggoner JJ, Abeynayake J, Balassiano I, Lefterova M, Sahoo MK, Liu Y, et al. Multiplex nucleic acid amplification test for diagnosis of dengue fever, malaria, and leptospirosis. J Clin Microbiol. 2014; 52(6): 2011-8.

2. Dahmana H, Granjon L, Diagne C, Davoust B, Fenollar F, Mediannikov O. Rodents as hosts of pathogens and related zoonotic disease risk. Pathogens. 2020; 9(3): 202.

3. Bounaadja L, Albert D, Chénais B, Hénault S, Zygmunt MS, Poliak S, et al. Real-time PCR for identification of *Brucella* spp.: a comparative study of IS711, bcsp31 and per target genes. Vet Microbiol. 2009; 137(1-2): 156-64.
